# Detection and Molecular Characterization of Rotavirus Infections in Children and Adults with Gastroenteritis from Vojvodina, Serbia

**DOI:** 10.3390/microorganisms10102050

**Published:** 2022-10-17

**Authors:** Aleksandra Patić, Vladimir Vuković, Gordana Kovačević, Vladimir Petrović, Mioljub Ristić, Milan Djilas, Petar Knežević, Tatjana Pustahija, Mirjana Štrbac, Jelena Djekić Malbaša, Smiljana Rajčević, Ivana Hrnjaković Cvjetković

**Affiliations:** 1 Department of Microbiology with Parasitology and Immunology, Faculty of Medicine, University of Novi Sad, 21000 Novi Sad, Serbia; 2 Institute of Public Health of Vojvodina, 21000 Novi Sad, Serbia; 3 Department of Epidemiology, Faculty of Medicine, University of Novi Sad, 21000 Novi Sad, Serbia; 4 Faculty of Sciences, University of Novi Sad, 21000 Novi Sad, Serbia; 5 Institute for Pulmonary Diseases of Vojvodina, 21204 Sremska Kamenica, Serbia

**Keywords:** rotavirus, gastroenteritis, genotyping, antigenic epitopes, vaccine, Serbia

## Abstract

Rotaviruses (RV) are the leading cause of gastroenteritis in infants, young children, and adults, responsible for serious disease burden. In the period 2012–2018, a cross-sectional study was conducted using stool samples collected from patients with acute gastroenteritis from Vojvodina, Serbia. We described age and gender distribution, as well as seasonal patterns of RV prevalence. Out of 1853 included stool samples, RV was detected in 29%. Hospitalized children between 1–2 years old were especially affected by RV infection (45%). The highest prevalence of infection was observed during the colder, winter/spring months. We compared sequenced representative G and P genotypes circulating in Serbia with vaccine strains and determined their genetic similarity. Genotype combination G2P[4] was the most prevalent (34.6%), followed by G2P[8] (24.1%) and G1P[8] (21.1%). Given that several epitopes were conserved, neutralization motifs among circulating strains can be characterized as sufficiently matching vaccine strains Rotarix™ and RotaTeq™, but existing antigenic disparities should not be overlooked. The present results contribute to a better insight into the prevalence of rotavirus infection in our region and point out the need for epidemiological surveillance of rotaviruses before the introduction of vaccines. These data can help formulate future vaccine strategies in Serbia.

## 1. Introduction

Viral gastroenteritis is one of the most common acute infectious diseases of people worldwide, and rotaviruses (RV) are the key viral pathogens responsible for severe gastroenteritis in infants and young children, but also in older adults (≥60 years) [1,2]. RV infections account for around 258 million cases of diarrhea in children younger than five years old, on a global level [1,3]. According to the reported estimates, they cause approximately 24 million outpatient visits, 2.4 million hospitalizations, and over 400,000 deaths per year [4,5]. In particular, RV is ranked as the third-leading pathogen associated with mortality in children below the age of five years [6].

Rotaviruses belong to the Reoviridae family. Two of their 11 genome segments encode the G (VP7) and P (VP4) proteins, which are used to classify rotaviruses into G (glycoprotein) and P (protease-sensitive) genotypes [1,7,8]. Currently, a total of 36 G and 51 P genotypes have been detected in both humans and animals around the world, with more than 60 G/P combinations identified in human populations, with the most common ones being G1P[8], G2P[4], G3P[8], G4P[8], G9P[8], and G12P[8] [1,8,9]. In recent years, a more detailed genetic classification based on all 11 genome segments has replaced the binary classification system [10]. Different strains show considerable regional variability in distribution, with some being globally more prevalent than what was previously thought [11]. Thus, it is especially important for rotavirus surveillance to include the identification of common strains in a particular geographical area and determine whether the most commonly circulating genotypes in the area are also those against which available vaccines provide immunity.

For the territory of Serbia and the surrounding countries, the data on the prevalence of rotavirus as a disease-causing pathogen are incomplete and inconsistent, which makes it difficult to assess its burden on populations. To effectively control RV infections, there is a need for quality epidemiological and clinical data, which would contribute to an assessment of the true disease burden and focus on preventive measures and early treatments. Currently, Serbia does not include rotavirus vaccines as mandatory but only as recommended vaccination for children [12].

In this study, we aimed to describe age and gender distribution and seasonal patterns, and to characterize the genotypes of rotavirus among children and adults with gastroenteritis from the province of Vojvodina, Serbia. The objective was to compare sequenced representative rotavirus G and P genotypes with vaccine strains and to determine their genetic similarity.

## 2. Materials and Methods

### 2.1. Study Design

This cross-sectional study was conducted from January 2012 to December 2018 at the Center for Virology of the Institute of Public Health of Vojvodina (IPHV), Novi Sad, Serbia. We included patients regardless of age (from one month old to over 90 years), in whom acute gastrointestinal syndrome of viral etiology was suspected. All participants included in the study were non-vaccinated against rotavirus. Stool samples were collected through the established collaboration of the IPHV with hospitals and healthcare centers on the territory of Vojvodina, Serbia. Criteria for the inclusion of patients were based on the WHO definition of acute gastroenteritis, as “an episode within 24h of at least three loose stools, and/or two or more vomiting episodes associated with diarrhea, in the 7 days before the medical visit; the episode must have been preceded by a symptom-free period of 14 days” [13]. Patients with a previously diagnosed chronic disease of the gastrointestinal tract (e.g., celiac disease, malabsorption, ulcerative colitis, Crohn’s disease), surgical disease of the gastrointestinal tract (e.g., intussusception, occlusions), recent abdominal surgery, cystic fibrosis, or food allergy with symptoms compatible with the definition of acute gastroenteritis, were excluded from the study. Informed consent was obtained from all participants prior to enrolment. For patients < 18 years old, informed consent was obtained from their parent(s) or legal guardian(s). Stool samples were screened for the presence of rotavirus using the real-time PCR kit (A.I.I. Screen Real-TM, Sacace Biotechnologies, Italy), according to the manufacturer’s instructions, and the results were also communicated to the treating physicians. A certain number of RV-positive samples (with a high copy number of RV genome) were selected for further analyses of genotyping or sequencing.

None of the authors were involved in the treatment of patients included, and all personal data were anonymized before being accessed and analyzed.

### 2.2. RNA Extraction, Detection, and Genetic Characterization of Rotavirus

For molecular analysis performed during the study, fecal samples were prepared as 10% suspensions in a balanced salt solution. About 150 µL of this suspension was used as the starting material for viral RNA extraction. RNA was extracted using either QIAamp Viral RNA Mini Kit (Qiagen, Milan, Italy) or Ribovirus Extraction Kit (Sacace, Biotechnologies, Italy) according to the manufacturer’s instructions.

### 2.3. Group A Rotavirus G Typing

Rotavirus group A (RVA) G typing was conducted using protocol from the previously described method [14]. For identification of G type, VP7-F and VP7-R or Beg9 and End9 consensus primers were used in first-round PCR. Amplicons of 881 base pairs (bp) or 1062 bp of gene 9 (VP7) were obtained. Second amplification was performed using the first PCR product as the template with G type-specific mixed primers aBT1, aCT2, mG3, aDT4, aAT8, mG9, mG10, and G12 reverse primer VP7R. Amplicons of 618 bp, 521 bp, 682 bp, 452 bp, 754 bp, 179 bp, 387 bp, and 266 bp were specifically generated for G1, G2, G3, G4, G8, G9, G10, and G12, respectively.

### 2.4. Group A Rotavirus P Typing

Group A rotavirus P typing was conducted using the protocol as previously described [14]. The first amplification of the VP4 gene was completed by RT-PCR using VP4F and VP4R primers. The second amplification was performed using a mixture of primers 2T-1, 3T-1, 1T-1D, 4T-1, 5T-1, P[11], and VP4F for the identification of P[4], P[6], P[8], P[9], P[10], and P[11] with amplicons of 362 bp, 146 bp, 224 bp, 270 bp, 462 bp, and 191 bp, respectively.

### 2.5. Agarose Gel Electrophoresis

Electrophoresis was performed for 60 min using 2% agarose gel. Separated amplicons were visualized using a UV spectrophotometer—Biometra Imaging System (Biometra, Germany). Images were taken and analyzed using the BioDocAnalyze software.

### 2.6. DNA Sequencing and Phylogenetic Analysis

A total of 10 RVA-positive stool samples with a high copy number of rotavirus genome were selected and prepared for direct Sanger sequencing. There were 10 representative samples obtained from six children (younger than 5 years) and four adult subjects, nine of which were from the hospital and one from an ambulatory setting. For the purpose of sequencing, the PCR products obtained in the first round of RVA genotyping were used. The PCR amplicons were purified with the QIAquick PCR Purification Kit (QIAGEN Inc., CA, USA) and sent to the Macrogen laboratory in the Netherlands for sequencing. The nucleotide sequences obtained of partial sequenced RVA, VP4 and VP7 genes were determined using the Basic Local Alignment Search Tool [BLAST] (http://blast.ncbi.nlm.nih.gov/Blast.cgi, accessed on 10 September 2022) and confirmed with the Virus Pathogen Database and Analysis Resource [ViPR]-tool (https://www.viprbrc.org/brc/rvaGenotyper.spg?method=ShowCleanInputPage&decorator=reo, accessed on 10 September 2022) [15]. Nucleotide sequences from the current study were previously submitted to GenBank with accession numbers MT786705-MT786722, OP270604, and OP270605.

For phylogenetic analysis, nucleotide sequences of the VP4 and VP7 genes of related strains were retrieved from GenBank together with the Rotarix™ and RotaTeq™ vaccine strains and aligned using the MUSCLE program within the MEGA version 6.06 software. The RVA G18P[17] pigeon strains for the VP4 gene (KX815053.1) and RVA pigeon G18P[X] for the VP7 gene (MG518479.1) were included in the phylogenetic trees as outgroups. DNA Model Test programs implemented in MEGA version 6.06 was used to identify the optimal evolutionary model. Using corrected Akaike Information Criterion (AICc) models T92 and T92+G+I were found to best fit the sequence data for the VP4 and VP7 genes, respectively. A phylogenetic tree was generated by the maximum likelihood method using the MEGA version 6.06 software with the Kimura2-parameter model and gamma distribution [16]. The robustness of branching was estimated with 1000 replicates. Alignment and comparison of nucleotide and deduced amino acid sequences of VP7 and VP4 genes were performed by using the BLAST web service and the ClustalW method.

### 2.7. Protein Structure Analysis of VP4 and VP7

We additionally studied amino acid changes within the antigenic epitope of the RVA outer-capsid proteins VP7 and VP4. The deduced amino acid sequences of the antigenic region of VP4 and VP7 proteins were compared with the corresponding strains of the currently licensed vaccines (Rotarix™ and RotaTeq™). Multiple amino acid alignments were carried out using the ClustalW ver. 2.1 software (http://bioedit.software.informer.com/download/?df3445, accessed on 10 September 2022). Structural analyses of VP7 and VP4 were performed using the UCSF Chimera molecular modeling system and Protein Data Bank (PDB) files 3FMG [17], and 1KQR [18]. Chimera was developed by the Resource for Biocomputing, Visualization, and Informatics at the University of California, San Francisco [19].

### 2.8. Statistical Analysis

We used descriptive statistics with absolute frequencies and percentage (%) to present data for categorical variables, and the chi-squared test or Fisher’s exact test (when only a few observations for individual cells were reported) was used to test differences in distributions between two groups. Results at the *p*-value <0.05 were considered statistically significant.

## 3. Results

### 3.1. Study Population

During the seven-year period, a total of 1853 stool samples were included in the study. A total of 53.7% were from male participants and the majority (32.5%) belonged to the age category 1–2 years, followed by 13.8% in the 3–5 years and 11.3% in the youngest age category, below 1 year old. Finally, 74.3% of all samples were collected among the hospitalized patients (Table 1).

### 3.2. Rotavirus Prevalence

Rotavirus was detected in 538/1853 (29%) of the analyzed stool samples. A higher percent (35.3%) was demonstrated in the hospitalized group in comparison to the ambulatory patients (10.9%) (*p* < 0.001), as presented in the Table 1.

**Figure 1:**
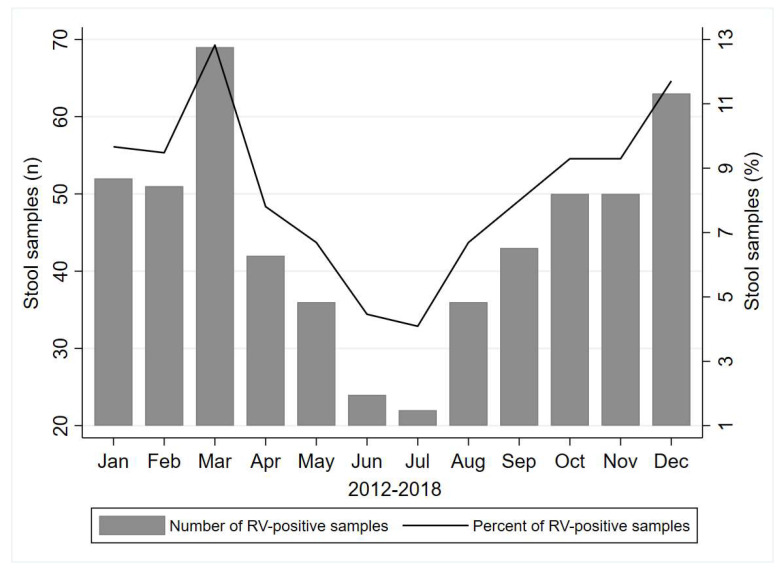
Number of RV-positive samples by month of registration, during the period 2012–2018 in Vojvodina province, Serbia.

There was a statistically significant difference between hospitalized and ambulatory-treated men and women with a higher percent of RV-positive samples from hospitalized patients (*p* < 0.001, for both sexes). Children of two years old demonstrated the highest proportion (45.5%) of rotavirus-positive samples in the hospitalized group, followed by 1 year old (45.4%) and 3–5 years old (40.5%), similarly as in the ambulatory patients with the highest percent in the 2 years old age category (23.6%) followed by children of the youngest age category (22.2%) and 1 year old (19.3%). When investigating the month of the year with the highest number of registered positive RV samples, we noticed the highest prevalence during the colder, winter/spring months, i.e., March with a total of 69 (12.8%) cases, followed by December (*n* = 63, 11.7%) and January (*n* = 52, 9.7%) during the period 2012–2018 years (Figure 1).

### 3.3. Distribution of G and P Genotypes of Group A Rotavirus

Genotyping of 133 RV-positive samples (with a high copy number of rotavirus genome) from different years of research was done. The number of genotyped samples was 95 from hospitalized patients and 38 from ambulatory patients. Among the G genotypes, G2 was the most predominant (58.6%), followed by G1 (22.6%) and G9 (6.8%) (Table 2). Aside from these single G genotypes, mixed G genotypes were also detected in 3.8% RV-positive samples. The G genotypes in mixed infections were G1 and G2, as well as G2 and G4. Two P genotypes were detected in the period between 2012 and 2018 in Vojvodina, Serbia, where the genotype P[8] was more frequent (63.2%) in respect to P[4] (36.8%). Great diversity was noticed in the combination of rotavirus G and P genotypes. Rotavirus genotype combination G2P[4] was the most prevalent (*n* = 46), followed by G2P[8] (*n* = 32) and G1P[8] (*n* = 28).

**Figure 2:**
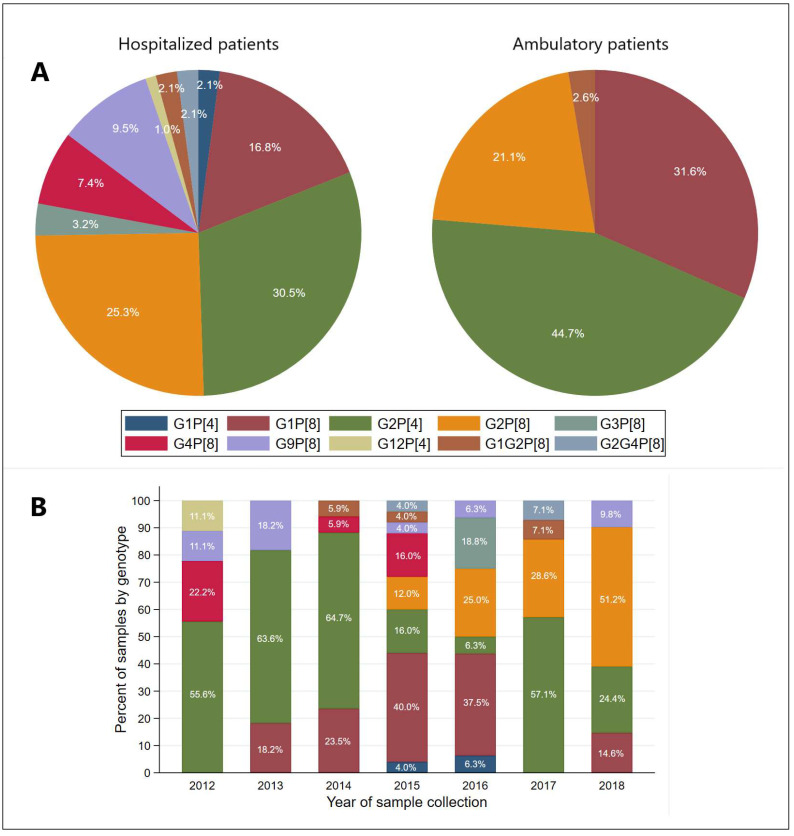
Rotavirus genotype distribution among the genotyped samples from hospitalized and ambulatory-treated patients (**A**) and yearly distribution of genotype by the year of sample collection (**B**).

Among hospitalized patients, the most prevalent genotype was G2P[4] (30.5%), followed by G2P[8] (25.3%) and G1P[8] (16.8%), while among the ambulatory treated patients, the majority of genotyped samples had genotype G2P[4] (44.7%), G1P[8] (31.6%), and G2P[8] (21.1%), as presented in the Figure 2A. When investigating the distribution of genotype by year of sample collection, we noticed that G2P[4] was detected every year and was the most prevalent in 2014 (64.7%), followed by 2013 (63.6%) and 2017 (57.1%) (Figure 2B). Genotype G2P[8] was detected in the highest percentage (51.2%) in 2018, followed by 28.6% in 2017 and 25.0% in 2016, while it was not among the genotyped isolates in 2012, 2013, and 2014. Genotype G1P[8] was detected every year, except for 2012 and 2017, with the highest percentage (40.0%) from samples collected in 2015 (Figure 2B).

**Figure 3:**
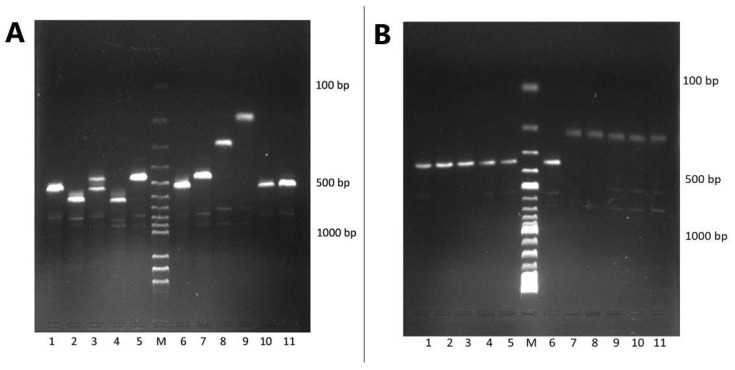
Rapid genotyping of human rotavirus: (**A**) G-typing multiplex PCR. Lanes: 1, 6, 10, and 11—G2 (521 bp); 2 and 4—G1 (618 bp); 3—mixed G2 and G4; 5 and 7—G4 (452 bp); M—DNA marker ladder; 8—G12 (266 bp); 9—G9 (179 bp); (**B**) P-typing multiplex PCR. Lanes: 1, 2, 3, 4, 5, and 6—P[4] (362 bp); 7, 8, 9, 10, and 11—P[8] (224 bp); M—DNA marker ladder.

Figure 3A,B shows the agarose gel of the representative amplified products of the G and P genotypes, respectively.

### 3.4. Phylogenetic Analysis and Comparison of the VP4 and VP7 Proteins of the RVA Strains with the Vaccine Strains

**Figure 4:**
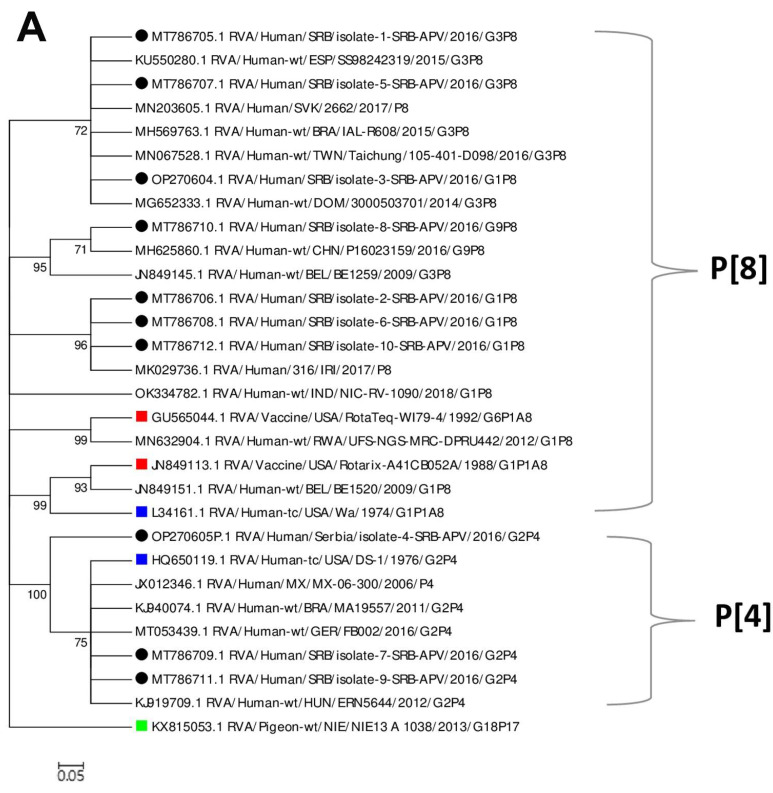

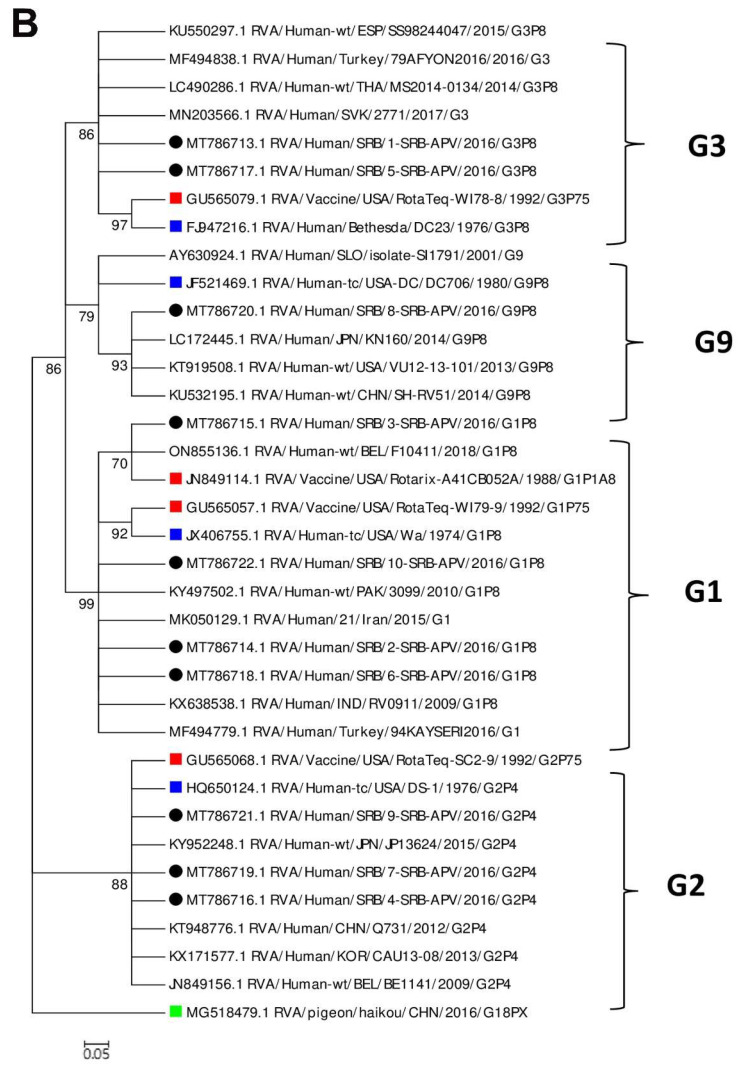
Phylogenetic relatedness of the rotavirus group A species based on VP4 gene (**A**), and VP7 gene (**B**). Phylogenies were reconstructed by the maximum likelihood method. The scale indicates nucleotide substitutions per position. Percentage bootstrap values above 70% are shown at branch nodes. Partial and complete sequences from other parts of the world and Rotarix™ and RotaTeq™ vaccine strains were included in the analysis. Key: The study sequences are indicated in black; vaccine strains in red; reference strains in blue; outgroups in green.

A total of 10 VP4 sequences of the partially sequenced gene 4 RVA (length about 590–600 bp) were analyzed. These sequences were compared to the sequences of currently available vaccines, Rotarix™ (JN84913) and RotaTeq™ (GU565044) together with the sequences identified in different geographic regions and reference strains Wa (L34161) and DS-1 (HQ650119). Generated phylogenetic trees are shown in Figure 4A,B, while Table S1 (Supplementary Materials) shows an alignment of the amino acid sequences of VP4 and VP7 rotavirus strains circulating in Vojvodina, Serbia with those of Rotarix™ and RotaTeq™ vaccines.

Based on the similarity to the reference isolate, it was determined that the study strains of RVA belong to P[8] and P[4] genotypes (Figure 4A). Further analysis between them and the RotaTeq™ vaccine P[8] and Rotarix™ vaccine P[8] strains revealed a moderate similarity according to their nucleic and amino acid sequences (nt, 97–99% and aa, 93–95%), and (nt, 95% and aa, 91%, respectively). A circulating VP4 P[4] strain had a slightly lower similarity with Rotarix™ (nt 84–86% and aa 75%) than with RotaTeq™ (84–85% for both nt and aa).

The RVA gene 9, which encodes VP7 protein was partially sequenced and sequences of 759–957 bp in length were analyzed. The phylogenetic tree was constructed for 10 sequences of VP7 protein together with sequences of isolates from different parts of the world that were downloaded from the GenBank database, including reference strains and strains of currently available vaccine sequences (Figure 4B). Amino acid sequences of study strains were analyzed together with the following vaccine strains: Rotarix™ (A41CB052A); RotaTeq (WI79-9) for G1; RotaTeq-SC2-9 for G2; RotaTeq-WI78-8 for G3; RotaTeq-BrB for G4; RotaTeq-WI79-4 for G6. Results are presented in Table S1 (Supplementary Materials). It was determined that the RVA study sequences belong to genogroup G1, G2, G3, and G9 which was confirmed based on the similarity with the reference isolates. The similarity of currently circulating G1 RVA with Rotarix™ strains ranged from 95.6% to 97.6%, and with RotaTeq™ (WI79-9) from 93.4 to 94.9%. Our isolates which belong to the G2 genogroup were rather distantly related to Rotarix™ strain, showing similarity from 73.5% to 74.2%. As expected, the VP7 of our RVA G2P4 and the G2 vaccine strain (SC2-9) component of RotaTeq™ were similar (approximately 95%). The VP7 proteins of the G3 RVA strains showed 81% and 93% of similarity to the VP7 component of Rotarix™ and RotaTeq™ (WI78-8), respectively. We identified only one RVA G9 strain in our samples which was relatively distantly related (79.5%) to VP7 of the Rotarix™ as well as to other strains of RotaTeq™ (77.7%–84.5%).

### 3.5. Comparison of the VP4 and VP7 Neutralizing Epitopes with Vaccine Strains

**Figure 5:**
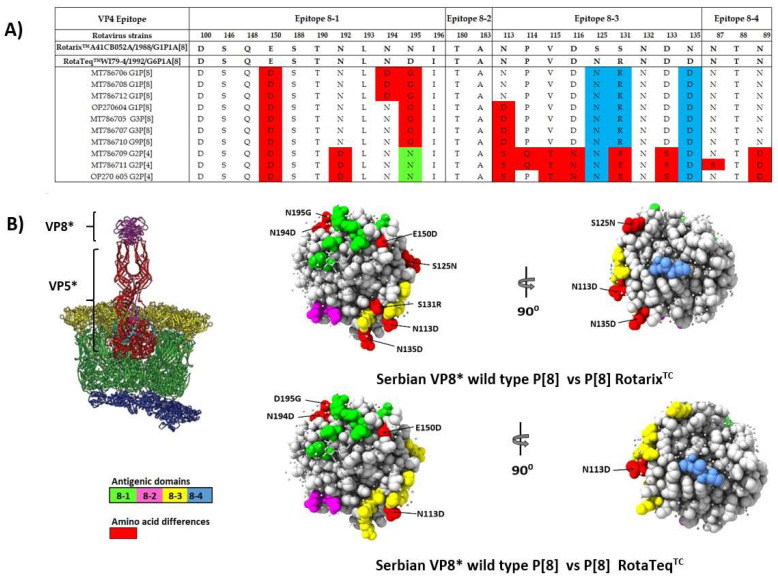
(**A**) Alignment of antigenic epitopes of the VP4 rotavirus strains circulating in Vojvodina, Serbia, with those of the Rotarix™ and RotaTeq™ vaccines. Alignment of the amino acid residues defining the neutralization domains of the VP4 protein (8-1, 8-2, 8-3, and 8-4) of rotavirus strains analyzed. (**B**) Surface representation of the globular domain VP8* of VP4 protein (Protein Data Bank (PDB) accession 1KQR). Antigenic epitopes 8-1, 8-2, 8-3, and 8-4 are marked in green, purple, yellow, and blue colors, respectively. Red color shows surface exposed residues which differ between our and vaccines strains.

The changes in the amino acid composition of the antigenic epitopes on the VP7 trimer and VP4 multimer (VP5* and VP8*) may reduce vaccine effectiveness. In that sense, we compared RVA strains from our study with those formulated by RotaTeq™ and Rotarix™ vaccines. The locations of these epitopes were presented by Dormitzer et al. (2002) and Aoki et al. (2009) [17, 18]. The VP4 spike protein contains 776 amino acids and after the proteolytic cleavage by trypsin it can be observed two fragments, designated VP8* (26 kDa) and VP5* (60 kDa). The VP8* subunit consists of four surface-exposed antigenic epitopes (8-1 to 8-4) while the VP5* subunit contains five antigenic regions, i.e., 5-1-5-5 [20]. Due to the partial sequencing of the VP4 gene (about 600bp), we were unable to perform comparisons at the level of VP5* antigenic epitopes, so we analyzed 25 residues of VP8* subunits (Figure 5A). All Serbian RVA VP4 strains had 13 of 25 residues which were identical to those in the VP4 antigenic epitopes of RotaTeq™ and Rotarix™. Epitopes 8-2 were completely conserved for all of our strains. Another characteristic of our strains was that all of them had E150D substitution. Regarding VP4 P[8] strains, the amino acid comparison showed that all Serbian isolates contain glycine at position 195 (epitopes 8-1). Thus, polar asparagine (N195G) in Rotarix™ and asparagic acid (D195G) in RotaTeq™ were replaced with non-polar glycine. Additionally, it was found that our P[8] strains contain approximately four amino acids different from antigenic epitopes in RotaTeq™ and seven amino acids different from those in Rotarix™. In general, more differences in amino acids were observed for the VP4 P[4] since it had twelve and eleven different amino acids compared with Rotarix™ and RotaTeq™, respectively. Mapping of these differences in the VP4 epitopes of the Serbian strains and the vaccine strains revealed that variations were distributed fairly heterogeneously across the front and back side of the molecule (Figure 5B).

**Figure 6:**
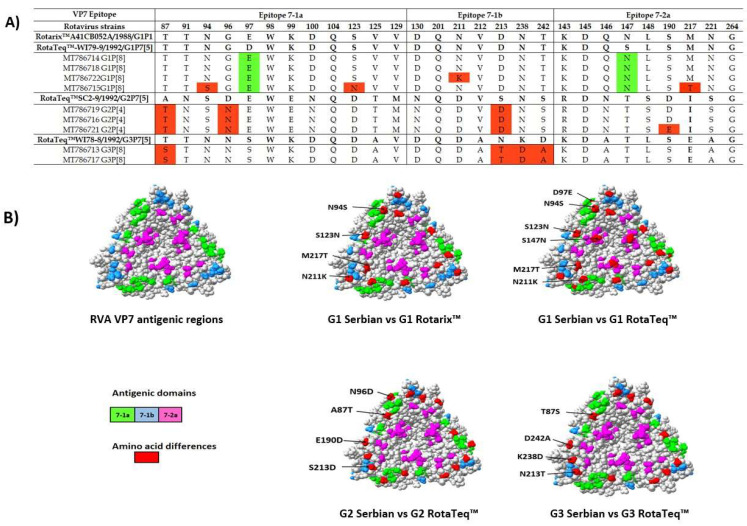
(**A**) Alignment of the amino acid residues in VP7 antigenic epitopes of the rotavirus strains circulating in Vojvodina, Serbia with those of the Rotarix™ and RotaTeq™ vaccines. Alignment of the amino acid residues defining the neutralization domains of the VP7 protein (7-1a, 7-1b, and 7-2a) of rotavirus strains analyzed. (**B**) Surface representation of VP7 trimer (Protein Data Bank (PDB) accession 3FMG). Antigenic epitopes 7-1a, 7-1b, and 7-2 are marked in green, blue, and purple colors, respectively. Red color shows surface exposed residues which differ between our vaccine strains.

We further analyzed the VP7 protein which has two critical antigenic epitopes: 7-1 (7-1a and 7-1b) and 7-2. We identified all sites within antigenic epitopes, except the amino acid position 291 in 7-1a. Results of the amino acid comparison between our circulating strains and analogue vaccine strains are presented in Table S1. Comparative analysis based on VP7 protein showed that our isolates contain a moderate to high percentage of amino acid differences in all of the subunits of VP7 epitopes (Figure 6A). Out of the 28 amino acid residues in VP7 neutralizing epitopes, only 16 (aa position: 91, 98, 99, 100, 104, 125, 129, 130, 201, 212, 143, 145, 146, 148, 221, and 264) were conserved among locally circulating Serbian strains compared to those used in vaccines. After the alignment of our study G1 RVA strains with the RotaTeq™ (WI79-9) of G1 and Rotarix™ vaccine strains, we identified a high level of similarity of amino acid residues between them. Interestingly, two G1 strains (MT786714, MT786718) had been shown to have almost identical VP7 antigenic epitopes as Rotarix™ vaccine strains, while two amino acid substitutions were found in comparison with G1 (position D97E and S147N) of RotaTeq™ (WI79-9). However, in the other two G1 strains from our study, four specific mutations in total were found (N211K, N94S, S123N, M217T).

There are few variations of amino acids in antigen epitopes for RVA G2 (A87T, S213D) compared to G2 of RotaTeq™. One of our G2 RVA strains had a single amino acid-specific mutation in position 190 (D190E). There were no differences between our VP7 G3 strains. However, they differed from analog vaccine strains in four amino acids, which is markedly more than in the case of G1 and G2 RVA strains. The only G9 RVA strain sequenced in this study differed in 8 out of 28 amino acids compared to Rotarix™. Mapping of the differences in the VP7 epitopes of the Serbian strains and the vaccine strains revealed that variations were distributed fairly heterogeneously and only on the front side of the molecule (Figure 6B).

## 4. Discussion

Viral gastroenteritis is one of the most common acute infectious diseases of people worldwide [1,8]. This study represents the first attempt to describe the age and gender distribution and seasonal patterns of the RV-caused gastroenteritis, to characterize the genotypes of RV among children and adults with gastroenteritis, and to determine their genetic similarity with the vaccine strains.

In the seven-year study period (2012–2018), rotavirus prevalence was the highest in 2015 (45.6%), followed by 34% in 2012, 30.1% in 2017, and 28.1% in 2016. Similar results were obtained in the studies conducted in developing countries in Central and Southeastern Europe [21], the Southeast Asian region [22], and Africa [23]. Causes of the high prevalence of RVA might be poor hygienic conditions, low infectious doses, high stability in the environment, presence of great genotypic diversity, and not including rotavirus vaccine in the routine pediatric immunization programs in Serbia [24].

A higher percent of rotavirus infection was demonstrated in the hospitalized group (35.3%) in comparison to the ambulatory patients (10.9%), which can be explained by a higher frequency of these infections in the youngest children, who have more severe clinical symptoms. Children between 1–2 years old demonstrated the highest proportion of rotavirus-positive samples in the hospitalized group, followed by 3–5 years old and 6–10 years old. Among the oldest respondents (>50 years), a higher percentage of rotavirus infections was found among hospitalized (32.8%) compared to ambulatory patients (7.5%). This can be explained by the fact that older patients are at a higher risk of adverse outcomes related to acute gastroenteritis, including the one caused by rotavirus. In some studies, there was a substantial diversity in RV prevalence among older adults, where RV positivity ranged from 1% to 15% [2]. Our research confirms the importance of rotavirus as a causative agent also among older adults.

Rotavirus infections occur primarily during cool, dry seasons, although the seasonality of rotavirus infections differs from one region to the other. Rotavirus is often referred to as “winter diarrheal disease” because in some parts of the world the majority of the cases are diagnosed in the winter season [1]. When considering the month of the year with the highest number of registered RV-positive samples in our study, we noticed the highest prevalence during the colder, winter/spring months. Our data regarding the seasonal pattern of disease occurrence are consistent with data obtained in other studies [25,26].

In our study, the genotyping of 133 rotavirus-positive samples from different years of research was done. Among the G genotypes, G2 was the most predominant (58.6%), followed by G1 (22.6%), G9 (6.8%), G4 (5.3%), G3 (2.3%), and G12 (0.8%). Slightly different results were obtained in a study conducted in several countries of Central and Southeastern Europe. The G1 was a predominant genotype in Slovenia (54.8%), Czech Republic (40.4%), and Croatia (24.2%), and G4 in Albania (46.2%) and Bulgaria (38.0%). Unlike Serbia, these countries had a lower prevalence of G2 type (3.0–11.5%), with the exception of Croatia (23.1%) [21].

Aside from single G genotypes, mixed G genotypes were also detected in 3.8% RV-positive samples. The G genotypes in mixed infections were G1 and G2, as well as G2 and G4. Mixed infections contribute to the emergence of novel rotavirus strains by providing a suitable environment for virus reassortment. Mixed G1 and G2 infections were also previously reported in Ireland, Italy, and Spain [27,28,29].

Two P genotypes were detected in the period between 2012 and 2018 in Vojvodina, Serbia. Rotavirus genotype P[8] was the most frequent (63.9%), followed by P[4] (36.1%), while other P genotypes were not detected. Results that match ours were reported in a study conducted across several countries of Central and Southeastern Europe, in which P[8] occurred most frequently (up to 80%) in all countries followed by P[4] (up to 35%). The P[1], P[6], and P[11] were rarely identified in Croatia and Slovenia [21]. Great diversity was detected in the combination of rotavirus G and P genotypes in our study. Rotavirus genotype combination G2P[4] was the most prevalent (34.6%), followed by G2P[8] (24.1%), G1P[8] (21.1%), G9P[8] (6.8%), G4P[8] (5.3%), G3P[8] (2.3%), G1P[4] (1.5%), and G12P[4] (0.8%).

“Common” G/P combinations, i.e., G1P[8], G2P[4], G3P[8], G4P[8], and G9P[8], accounted for 82.6% of the cases in Bulgaria, 77.7% in the Czech Republic, and 71.2% in Slovenia. The most prevalent overall was G1P[8] (32.1%), followed by G4P[8] (18.5%) and G2P[4] (8.1%), while G9P[8] (5.2%) and G3P[8] (2.2%) were found less frequently. “Uncommon” G/P combinations were quite prevalent in Albania, where they accounted for 23.4% of cases overall, with G4P[4] detected in 13.3%, G1P[4] in 4.4%, and G2P[8] in 5.7% of the cases [21]. “Uncommon” G/P combinations (G2P[8], G1P[4], and G12P[4]) were also found in our study in a noticeable percentage. 

Distribution patterns of commonly detected RV genotypes can vary by year, country, and age [30]. Both in Belgium and the UK, prior to vaccine introduction, G1P[8] was the most common circulating genotype, while following vaccination G2P[4] has been detected more frequently [31,32]. In the pre-vaccine period in Italy, G1P[8] was also the prevalent RV genotype accounting for 62.4% of the cases, while after the introduction of the vaccine, a variety of prevalent RV genotypes have been observed, including G2P[4] in 2015 and 2017, G9P[8] in 2016–2017, G3P[8] in 2018–2019, and G12P[8] in 2020 [33]. Increasing data suggest that the changes may be due to the impact of strain-specific vaccines [34]. However, changes in genotype distribution also occur in countries that have not introduced vaccination [35], so it is not clear whether the vaccine directly leads to a change in genotype diversity [36]. In our study, the most frequent genotypes also changed during the years of research. Genotype G2P[4] was detected every year in our study and was the most prevalent in 2014 (64.7%), followed by 2013 (63.6%) and 2017 (57.1%). As for the other most prevalent genotypes, G2P[8] was detected in the highest percentage (51.2%) in 2018, while G1P[8] was in the highest percentage (40.0%) in the samples collected in 2015.

The diversity of rotavirus genotypes and strains prevalent in different areas of the world provides valuable insight into rotavirus evolution. There are three major mechanisms that, both individually and in combination, shape the evolution of human rotaviruses and contribute to their diversity [22,37]: point mutations (singular or accumulated over time), genomic reassortment after a mixed infection of a single cell, and introduction of animal rotaviruses into the human population [37,38].

Data obtained from the current study show that RV is a common cause of acute gastroenteritis in the investigated population. More than 10 years after the authorization of two rotavirus vaccines [39], these vaccines are still not mandatory in the Republic of Serbia, but are recommended for infants at the age of 2 months and older. Countries with routine RV immunization programs, such as the USA, Australia, Brazil, and Mexico have achieved reductions in the number of infants and children needing hospitalization or emergency department care for RV disease by about 85% [40]. Regardless of the fact that vaccines are effective against the commonly detected human RVA strains, vaccine-induced immunological pressure might result in the selection of more resistant strains [41]. In the present study, we compared the VP4 and VP7 of 10 locally circulating RVAs with antigenic epitopes of the two licensed, and available, RVA vaccines in Serbia, namely, Rotarix™ and RotaTeq™. By comparative analysis of their VP4 and VP7 regions, we identified possible important antigenic disparities that could potentially reduce vaccine effectiveness.

The VP4 spike protein is involved in several important structural and functional roles such as virus particle binding, penetration, and maturation, making this protein very important in viral neutralization. In our study, dominating strains were VP4 P[8] and P[4]. All of our VP4 strains had the E150D substitution which was the change identified relative to the both RotaTeq™ and Rotarix™ vaccine analogue strain. Aside from that, another amino acid difference was located on the surface of the protein structure at position 195 (Asn/Asp to Gly). The amino acid changes identified at positions N113D, S131R and N135D in this study are previously described by a few search groups from Rwanda [42] and Zambia [43]. They resulted in polarity changes and play a role in RV’s escape from host immunity.

The alignment of the deduced amino acid sequences of the VP7 genes revealed that our circulating G1 and G2 strains show satisfactory match with the antigenic VP7 epitopes of vaccine Rotarix™, while up to two amino acid substitutions have been seen with RotaTeq™. Compared with the VP7 G1 component of RotaTeq™ vaccine, all of Serbian G1 strains had an amino acid substitution at position 97 (Asp to Glu) and 147 (Ser to Asn) which refer to differences in the electrostatic charge distribution. These variations are known neutralization escape mutation sites and have been described among G1 strains around the world [42,43]. Additionally, few single-point mutations occurred in the other two G1 strains in the study. Some of these substitutions (N94S, S123N, N211K, and M217T) have previously been seen among the Iranian and Lebanese G1 strains [44,45]. Since the application of RV vaccines in Serbia is currently not widespread, the discovery of these variants in our country indicates the possibility that they were imported and did not appear locally under the pressure of a selective vaccine.

The G2 strains were the most frequently detected VP7 RVA strains in the current study. Such results are not surprising because G2 strains have been rapidly evolving and spreading during the last decade, especially after the implementation of the vaccine. Since G2 RVA strains are generally associated with P[4] genotype, protection against G2 RVA strains afforded by RotaTeq™ mainly depends on the G2 component of the vaccine [20]. On the other hand, G3 strains were relatively rarely detected during the study period. Although, both of sequenced G3 strains displayed an intragenotypic homogeneity, they were distanced from the analog and RotaTeq™ (WI78-8) G3 strain. One of our RVA G3 strain had K238N/D amino acid substitutions previously identified among Belgian [20], Argentinean [46] and Iranian [44] strains. It is well known that these mutations are associated with a potential N-linked glycosylation site [20], which would prevent neutralizing antibody activity [47].

Our study provided important information on the genetic and antigenic properties of the Serbian and the vaccine strains before the RVA vaccine implementation in our country. We performed a protein modeling analysis of VP7 and VP4 in order to observe the sites of mutations within their immunodominant segments. The majority of the variations found among our strains were changes in amino acid charge and polarity. However, one of our RVA G3 strains had a K238N/D amino acid substitution associated with a potential N-linked glycosylation site. The change in charge may affect the protein’s chemical properties, while the shift in polarity suggests possible inaccessibility of the epitope as it becomes more hydrophobic [48]. To understand whether the observed changes in electrical charges affect antigen–antibody binding and lead to loss of vaccine-induced protection, additional studies must be conducted. Protein modeling analyses showed that certain amino acid differences were found at more exposed residues than others, increasing the chance of the virus to escape neutralizing antibodies. Overall, neutralization motifs among circulating strains in the investigated area can be characterized to sufficiently match vaccine strains (Rotarix™ and RotaTeq™), given that several epitopes were conserved. In addition, the broad cross-protective activity of RVA vaccines and the proven and recognized vaccine’s effectiveness around the world should not be overlooked [39,42,43].

One of the limitations of this study was that the sequencing of the gene segments RVA VP7 and RVA VP4 was partial. Although this data was suitable to discriminate RVA genotypes, it could not distinguish among subgenotypic lineages. In recent times, there has been substantial evidence that subgenotypic lineages differ in antigenic properties, potentially allowing some RVA strains to escape adaptive immunity [20,49]. Due to a partial sequencing of the VP4 gene (about 600 bp), we were unable to perform an analysis of VP5* antigenic epitopes. Despite that, this study provided significant findings of genetic and possible antigenic differences at the level of immunodominant segments of the VP7 and VP4 genes between the circulating strains of RVA in our region and their relation to the analogue genes in Rotarix™ and RotaTeq™ vaccine strains. This study provided preliminary data for further research, which could include sequencing of the whole RVA genome. Finally, for a better understanding of the immune defense against rotaviruses, genetic monitoring of other RVA genes would also be important.

## 5. Conclusions

In summary, rotavirus infections are responsible for a substantial proportion of acute gastroenteritis cases among Serbian patients, where children aged less than five years are especially affected. The study demonstrated a high prevalence of genotypes G2 (58.6%) but mixed G genotypes were also detected in 3.8% of rotavirus-positive samples. Neutralization motifs in circulating strains suggest a sufficient match with vaccine strains of Rotarix™ and RotaTeq™. On the other hand, the results of the current study indicate the existence of potential antigenic disparities in circulating strains compared to Rotarix™ and RotaTeq™, which can be of importance in the context of the expected wider application of these vaccines. Presented data highlight the need for robust surveillance of rotavirus to formulate future vaccine strategies in our country. Considering the fact that dynamic changes in rotavirus genotypes cannot be excluded, careful monitoring of the emergence of unusual RVA strains is highly recommended. These data cannot be a definitive answer on RV vaccine effectiveness, but they could be a relevant starting point for national and regional health authorities in initiating additional research on rotavirus in our country, which could lead to a creation of an optimal health policy in order to reduce RVA infections.

## Figures and Tables

**Table 1:** Demographic characteristics of patients with RV-positive stool samples during the period 2012–2018 in Vojvodina province, Serbia, by hospitalization status.

	Total, Positive/Tested (%)	Hospitalized Patients, Positive/Tested (%)	Ambulatory Patients, Positive/Tested (%)	*p*-Value ^1^
**Total**	538/1853 (29.0)	486/1376 (35.3)	52/477 (10.9)	**<0.001**
**Sex, *n* (%)**				
Male	292/995 (29.3)	265/733 (36.2)	27/262 (10.3)	**<0.001**
Female	246/858 (28.7)	221/643 (34.4)	25/215 (11.6)	**<0.001**
**Age category (years), *n* (%)**				
<1	62/209 (29.7)	56/182 (30.8)	6/27 (22.2)	0.364
1	164/394 (41.6)	153/337 (45.4)	11/57 (19.3)	**<0.001**
2	83/209 (39.7)	70/154 (45.5)	13/55 (23.6)	**0.005**
3–5	87/256 (34.0)	83/205 (40.5)	4/51 (7.8)	**<0.001**
6–10	44/189 (23.3)	40/128 (31.3)	4/61 (6.6)	**<0.001**
11–15	20/128 (15.6)	16/91 (17.6)	4/37 (10.8)	0.428
16–20	15/110 (13.6)	14/89 (15.7)	1/21 (4.8)	0.294
21–30	19/102 (18.6)	18/71 (25.4)	1/31 (3.2)	**0.011**
31–40	12/73 (16.4)	11/37 (29.7)	1/36 (2.8)	**0.003**
41–50	6/42 (14.3)	5/21 (23.8)	1/21 (4.8)	0.184
>50	26/141 (18.4)	20/61 (32.8)	6/80 (7.5)	**<0.001**

^1^ Pearson’s chi-squared test or Fisher’s exact test (when only few observations for individual cells were reported). Significance levels are given in bold for *p* < 0.05.

**Table 2:** Distribution of rotavirus G and P genotypes among children and adults with gastroenteritis in Vojvodina, Serbia during the period 2012–2018 years.

G Genotypes	P Genotypes, *n* (%)		
	P[4]	P[8]	Total
**G1**	2 (4.1)	28 (33.3)	30 (22.6)
**G2**	46 (93.9)	32 (38.1)	78 (58.6)
**G3**	0	3 (3.6)	3 (2.2)
**G4**	0	7 (8.3)	7 (5.3)
**G9**	0	9 (10.7)	9 (6.8)
**G12**	1 (2.0)	0	1 (0.7)
**G-mixed**	0	5 (6.0)	5 (3.8)
Total	49 (100)	84 (100)	133 (100)

## Data Availability

The data that support the findings of this study are available from the corresponding author upon reasonable request.

## Supplementary Materials

The following supporting information can be downloaded at: https://www.mdpi.com/article/10.3390/microorganisms10102050/s1, Table S1: Alignment of the amino acid sequences of the VP4 and VP7 rotavirus strains circulating in Vojvodina, Serbia with those of the Rotarix™ and RotaTeq™ vaccines.
