# Supplementary material for: Detection and Molecular Characterization of Rotavirus Infections in Children and Adults with Gastroenteritis from Vojvodina, Serbia
Source: Microorganisms. 2022 Oct 17;10(10):2050. doi: 10.3390/microorganisms10102050 (PMC9607116; doi:10.3390/microorganisms10102050)
Supplement: Supplementary file 1 [file microorganisms-10-02050-s001.zip › microorganisms-1917677-supplementary.pdf]

Table S1. Alignment of the nucleotide sequences of the VP4 and VP7 rotavirus strains circulating in Vojvodina, Serbia with those of the Rotarix™ and RotaTeq™ vaccines.

SEQUENCE VP4			SEQUENCE VP7						
STRAIN	Rotarix G1P1A[8] [%]	RotaTeq G6P1A[8] [%]	STRAIN	Rotarix G1P[8] [%]	G1 RotaTeq [%]	G2 RotaTeq [%]	G3 RotaTeq [%]	G4 RotaTeq [%]	G6 RotaTeq [%]
MT786706 G1P[8]	91.33	93.37	MT786714 G1P[8]	96.77	93.91	73.74	78.14	74.91	79.57
MT786708 G1P[8]	91.46	93.47	MT786718 G1P[8]	97.62	94.86	72.73	79.84	78.80	79.84
MT786712 G1P[8]	91.33	93.37	MT786722 G1P[8]	96.82	94.35	76.07	81.27	78.80	80.92
OP270604 G1P[8]	89.32	94.66	MT786715 G1P[8]	95.61	93.42	75.24	79.62	76.80	80.88
MT786707 G3P[8]	90.26	94.36	MT786717 G3P[8]	80.70	80.38	76.90	92.41	76.90	85.76
MT786705 G3P[8]	89.32	94.44	MT786713 G3P[8]	81.0	80.69	76.95	92.89	77.26	86.29
MT786710 G9P[8]	90.26	93.33	MT786720 G 9P[8]	79.50	78.78	76.90	84.53	77.70	82.73
MT786709 G2P[4]	82.65	84.13	MT786719 G2P[4]	74.20	75.16	95.54	74.52	71.02	75.16
MT786711 G2P[4]	82.14	83.67	MT786721 G2P[4]	73.51	74.50	95.36	74.50	70.53	74.83
OP270605 G2P[4]	83.92	85.43	MT786716 G2P[4]	74.13	74.76	94.95	74.13	70.98	74.76

Similarity percentages with analog-examined Rotarix™ vaccine strains higher than 90% are colored in blue; similarity percentages with analog-examined RotaTeq™ vaccine strains higher than 90% are colored in red.
